# Retraction: Role of high expression levels of STK39 in the growth migration and invasion of nonsmall cell type lung cancer cells

**DOI:** 10.18632/oncotarget.28881

**Published:** 2026-05-13

**Authors:** Zhao Li, Wenzhuo Zhu, Liwen Xiong, Xiaobo Yu, Xi Chen, Qiang Lin

**Affiliations:** ^1^Department of Thoracic Surgery, Shanghai General Hospital, Shanghai Jiaotong University School of Medicine, Shanghai, China; ^2^Department of Pulmonary Diseases, Shanghai Chest Hospital, Shanghai Jiaotong University School of Medicine, Shanghai, China; ^*^These authors contributed equally to this work

**This article has been retracted:** Oncotarget has concluded its investigation of this paper, and the following issues were discovered:

- Figure 3C: The NCI-H358/STK39 western blot is a duplicate of a blot in Figure 5A (A549/p-AKT) of a concurrently published and retracted article [1]. Additionally, the GAPDH/NCI-H358 and GAPDH/NCI-H1975 western blots are duplicates.
- Figure 4B: The flow cytometry plots for NCI-H1975/siNC and A549/WT are duplicates.
- Figure 6A: The E-cadherin blot image is a duplicate of the MMP2 image found in Figure 7C of another concurrently published and retracted paper [2]. Furthermore, the NCI-H1975/PCNA and NCI-H1975/CDC25A western blots are duplicates.

External Duplications: Several images were found in unrelated papers published later:

- The Figure 3A STK39 western blot was duplicated as a TBX2 blot in Figure 2A of reference [3] and as CXCR5 in Figure 2C of reference [4].
- The A549/p38 western blot bands in Figure 6B were found in Figure 5A as MG63/JAK2 in reference [5].

The authors did not respond to requests for comment or provide the original data. Consequently, the editorial decision has been made to retract this paper. Oncotarget has reached out to all authors to notify them of this retraction but has received no response.

Original article: Oncotarget. 2016; 7:61366–61377. 61366-61377. https://doi.org/10.18632/oncotarget.11351

## References

[1] Wang X, Shi W, Shi H, Lu S, Wang K, Sun C, He J, Jin W, Lv X, Zou H, Shu Y. TRIM11 overexpression promotes proliferation, migration and invasion of lung cancer cells. J Exp Clin Cancer Res. 2016; 35:100. 10.1186/s13046-016-0379-y. 27329103. Retraction in: J Exp Clin Cancer Res. 2023; 42:139. DOI: 10.1186/s13046-023-02721-1 PMID: 37264372 PMC4915141

[2] Huang Z, Li J, Du S, Tang Y, Huang L, Xiao L, Tong P. FKBP14 overexpression contributes to osteosarcoma carcinogenesis and indicates poor survival outcome. Oncotarget. 2016; 7:39872–84. 10.18632/oncotarget.9524. 27223089. Retraction in: Oncotarget. 2025; 16:131. DOI: 10.18632/oncotarget.28697 PMID: 40019484 PMC5129977

[3] Lv Y, Si M, Chen N, Li Y, Ma X, Yang H, Zhang L, Zhu H, Xu GY, Wu GP, Cao C. TBX2 over-expression promotes nasopharyngeal cancer cell proliferation and invasion. Oncotarget. 2017; 8:52699–707. 10.18632/oncotarget.17084. 28881763 PMC5581062

[4] Chen W, Wang Y, Zhou T, Xu Y, Zhan J, Wu J. CXCL13 Is Involved in the Lipopolysaccharide-Induced Hyperpermeability of Umbilical Vein Endothelial Cells. Inflammation. 2020; 43:1789–96. 10.1007/s10753-020-01253-6. 32500306 PMC7476967

[5] Zhang S, Wang Y, Chen S, Li J. Silencing of cytoskeleton-associated protein 2 represses cell proliferation and induces cell cycle arrest and cell apoptosis in osteosarcoma cells. Biomed Pharmacother. 2018; 106:1396–403. 10.1016/j.biopha.2018.07.104. 30119212

